# Differences in Median Ultraviolet Light Transmissions of Serial Homeopathic Dilutions of Copper Sulfate, *Hypericum perforatum*, and Sulfur

**DOI:** 10.1155/2013/370609

**Published:** 2013-01-15

**Authors:** Sabine D. Klein, Annegret Sandig, Stephan Baumgartner, Ursula Wolf

**Affiliations:** ^1^Institute of Complementary Medicine (KIKOM), University of Bern, 3010 Bern, Switzerland; ^2^Society for Cancer Research, Hiscia Institute, 4144 Arlesheim, Switzerland

## Abstract

Homeopathic remedies are produced by potentising, that is, the serial logarithmic dilution and succussion of a mother tincture. Techniques like ultraviolet spectroscopy, nuclear magnetic resonance, calorimetry, or thermoluminescence have been used to investigate their physical properties. In this study, homeopathic centesimal (c) potencies (6c to 30c) of copper sulfate, *Hypericum perforatum*, and sulfur as well as succussed water controls were prepared. Samples of these preparations were exposed to external physical factors like heat, pressure, ultraviolet radiation, or electromagnetic fields to mimic possible everyday storage conditions. The median transmissions from 190 nm to 340 nm and 220 nm to 340 nm were determined by ultraviolet light spectroscopy on five measurement days distributed over several months. Transmissions of controls and potencies of sulfur differed significantly on two of five measurement days and after exposure to physical factors. Transmissions of potencies exposed to ultraviolet light and unexposed potencies of copper sulfate and Hypericum perforatum differed significantly. Potency levels 6c to 30c were also compared, and wavelike patterns of higher and lower transmissions were found. The Kruskal-Wallis test yielded significant differences for the potency levels of all three substances. Aiming at understanding the physical properties of homeopathic preparations, this study confirmed and expanded the findings of previous studies.

## 1. Introduction

Homeopathic preparations (hp) are used in complementary medicine worldwide, but homeopathy has been and is still vigorously debated [1–4], and these debates are based on prior believes [5]. While there seems to be good preclinical and clinical evidence for specific effects of hp [6–15], the underlying mode of action is yet unclear. Our aim is to determine potential physical properties of hp, which eventually may allow a scientific understanding of hp.

Homeopathic remedies are produced by potentising, that is, the serial logarithmic dilution and succussion of a mother tincture. Several standard techniques of measuring physical properties of hp have been used in previous studies [16], including ultraviolet (UV) spectroscopy [17–24], nuclear magnetic resonance techniques [25–31], calorimetry [32], and thermoluminescence [33, 34].

In preceding studies, we observed significant differences in the transmission of UV light between hp and controls [23, 24], and between hp exposed to physical factors and unexposed hp [23]. The aim of the present study was (i) to target reproducing our results and (ii) to expand the nature of starting materials and external physical factors. We included a dilution of Hypericum perforatum, a plant often used in homeopathy, anthroposophically extended medicine, and phytotherapy. Since many questions about the stability of hp remain unsettled, for example, regarding storage conditions, sterilisation procedures, or exposure to radiation from mobile phones and scanners, we exposed hp to elevated temperature, pressure in an autoclave, UV light, and non-ionising radiation.

## 2. Materials and Methods

### 2.1. Materials

Hp were prepared from copper sulfate (CuSO_4_; Weleda AG, Arlesheim, Switzerland), sublimed sulphur (S_8_; Phytomed AG, Hasle/Burgdorf, Switzerland), and Hypericum perforatum alcoholic dilution mother tincture, 62% alcohol (hypericum; Herbamed AG, Bühler, Switzerland).

Cleaning of the vessels for potentisation, autoclavation and rinsing the tubes and cuvettes during the measurements was performed by using 18 MΩ distilled sterile water (purified water by Arium 61316 reverse Osmosis System, Satorius Stedim AG, Aubagne, France). For the preparation of controls and hp, 18 MΩ autoclaved distilled deionised water (Hiscia Institute, Arlesheim, Switzerland) was used, delivered in 10 L Schott Duran bottles (VWR International Dietikon, Switzerland).

All of the hp and controls were stored and potentised in 500 mL narrow necked bottles with standard ground joint and a conical shoulder, made from borosilicate glass with hydrolytic class 1, that is, highly resistant against corrosion in neutral, basic, and acid environments (Schott Duran, VWR International Dietikon, Switzerland), closed with standard ground Duran flat-head stoppers. 

Potentisation vessels and stoppers as well as UV measurement test tubes were reused from former experiments [23, 24]. Preceding potentization all vessels were cleaned by rinsing three times with 18 MΩ water in order to decrease potential ion leaching from the vessel wall. The same procedure was applied to the test tubes used in the autosampler of the UV-spectrometer (see below). The process of cleaning, drying, and filling all vessels and test tubes was performed in a laboratory under laminar flow (Prettl GmbH, Pfullingen, Germany). The test tubes for UV measurements of the samples were 18 mL tubes, made from hydrolytic glass (Schott Fiolax, Mitterteich, Germany) and were filled with the homeopathic samples by one-way 20 mL, sterile, polystyrene pipettes (Pipetboy acu, Integra Bioscience AG, Zizers, Switzerland).

### 2.2. Sample Preparation

The hp were prepared according to the legal regulation for homeopathic remedies [35] by using the multiple glass method. Potentisation was performed by hand through horizontally shaking the vessel at a rate of about 2.7 Hz for 4 min for CuSO_4_ and S_8_, and for 2.5 min for hypericum prior to each dilution step.

All hp were made as c preparations (i.e., centesimal potency means 100-fold dilution with each step) up to 30c. CuSO_4_ and hypericum had each 10 independent succussed water controls, while S_8_ had 12 controls, prepared with each 5 (6) vessels before and 5 (6) after the potentisation process to examine possible cross contamination. Controls were produced by shaking the potentisation medium (water) at the same duration as the hp, but controls were not diluted.

All steps of preparation and handling with open vessels were performed under a laminar flow box wearing sterile examination gloves and a lab coat to prevent unwanted contamination of the samples. Vessels were shielded with aluminium foil and stored closed in boxes at stable temperature and humidity.

Computer-generated random codes were used for randomisation. Blinding of the vessels was performed by an unbiased person, lists of the allocation of contents to the vessels were kept closed until the end of data attainment and data reduction.

### 2.3. Exposure to External Physical Factors

Samples of hp and controls were exposed to one of the following external physical factors: (1) incubation (Incubator, Sauter, Switzerland) at 37°C for 24 hours, (2) UV light at 252 nm of a sterilisation lamp for 12 hours (CAMAG Reprostar, Switzerland), (3) heat under pressure by autoclave (Fedegari Autoclavi, Vitaris AG, Baar, Switzerland) at a temperature of 90°C for 20 minutes, filled into autoclavable Duran vessels (Schott Fiolax), or (4) an electromagnetic field of a mobile phone (Philips, Savvy Dual Band) at 900 MHz with an output of 2 W for 120 minutes while the test tubes with samples were placed on a turning plate under the laminar flow.

### 2.4. UV Spectroscopy

Data were acquired by a Shimadzu UV PC 1650 spectrometer (Kyoto, Japan) with a wavelength range from 190 to 1100 nm, equipped with an auto sampler CETAC ASX-260 (Omaha, USA), and a sipper. 

Comprehensive preparatory measurements were achieved in previous investigations to identify the impact of instrumental parameters on reproducibility such as wavelength of lamp change from visible (VIS) to UV lamp and scan speed, instrumental drift, warm-up time, number of repetitions, sip and purge time. Prior to the measurements, a baseline calibration was completed with the cuvette filled with 18 MΩ water. Light transmission was measured from 190 to 1100 nm. Each measurement was repeated four times with the first run including five samples of 18 MΩ water as a run-in before the actual samples. The unit of equipment was engaged 10 hours prior to actual measurements to achieve an efficient warm-up and to decrease the instrumental drift. Room temperature and humidity were kept constant. Heated samples were allowed to regain room temperature before the measurements.

**Figure 1:**
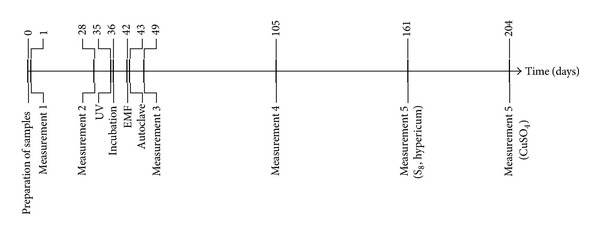
Timeline of preparation of the samples and measurements. After 34, 35, 41, and 42 days, samples of potencies and controls were either exposed to UV light (UV) for 12 h, incubated at 37°C for 24 h (incubation), exposed to an electromagnetic field (EMF) for 2 h, or incubated in an autoclave (autoclave) for 20 min, respectively, and light transmission was measured the following day (days 35, 36, 42, and 43, resp.).

Figure 1 shows the timeline of preparation of the samples and measurements.

### 2.5. Data Analysis

To compare measurements performed on different days, the common daily variations of a UV spectrophotometer that occur due to a new calibration on each measurement day had to be corrected for. Thus, transmissions of the samples (controls or hp) were divided by transmissions of the pooled controls for each day and wavelength (nm).

Median transmission values were calculated for the ranges of 190 nm–340 nm and 220 nm–340 nm as in [23]. Since not all data were normally distributed, non-parametric tests (Mann-Whitney-*U*, Kruskal-Wallis and Jonckheere-Terpstra) were used to compare controls and hp on the same measurement day or hp on different measurement days. Effect sizes (*r*) were calculated and results were reported according to [36].

In order to compare the present study to the previous ones performed by our group [23, 24], differences of means (transmission of controls − transmission of hp) in % and 95% confidence intervals were calculated. All measurements of CuSO_4_ and of S_8_ were finally combined with the number of measurement days as weight.

SPSS Statistics 17.0 and 20.0 (IBM, Armonk, USA) was used for statistical analyses.

## 3. Results

### 3.1. Controls Prepared before and after the hp

When controls 1 (prepared before the series of potencies) and controls 2 (prepared after the series of potencies) were compared by Mann-Whitney-*U* test, only in measurement 1 (of 5) of the CuSO_4_ measurements a statistically significant difference was found, but in none of the measurements of hypericum or S_8_. Therefore, it was concluded that the order of preparation did not have an effect on the transmissions measured, and, consequently, controls 1 and 2 for each substance were combined for further calculations.

### 3.2. Differences between Controls and hp

Controls and hp of each measurement day were compared separately. In unexposed samples, controls and hp differed significantly for S_8_ on measurement days 2 and 3 (Table 1). While controls of CuSO_4_ and hypericum tended to have higher transmissions than hp, controls of S_8_ had lower transmissions than hp.

Controls and hp of samples exposed to physical factors showed significant differences in transmission for CuSO_4_ after incubation, for hypericum after exposure to UV, and for S_8_ after all of the 4 factors (Table 1). Both ranges of transmission (190 nm–340 nm and 220 nm–340 nm) yielded similar results with respect to significant differences between groups.

### 3.3. Influence of Ageing

To investigate the possible influence of ageing on hp, measurements 1 to 5 of hp (without controls) were compared using a Kruskal-Wallis test. Significant differences between the 5 measurement days were found for S_8_ (190 nm–340 nm: *P* = 0.002; 220 nm–340 nm: *P* = 0.004). Jonckheere's test revealed no significant trend in the data. For CuSO_4_ and hypericum, no differences between the measurement days were found.

### 3.4. Effect of Exposure to External Physical Factors

It was investigated whether exposure of the hp to external physical factors had an effect on transmission compared to non-exposed hp. For that purpose, non-exposed hp of measurements 2 and 3 were combined and compared to exposed hp (Table 2). Significant differences were found for CuSO_4_ after incubation and UV as well as for hypericum after UV, where transmissions of hp after exposure to these physical factors were reduced compared to transmissions of non-exposed hp. No significant changes have been observed for S_8_.

### 3.5. Differences between Potency Levels

**Figure 2:**
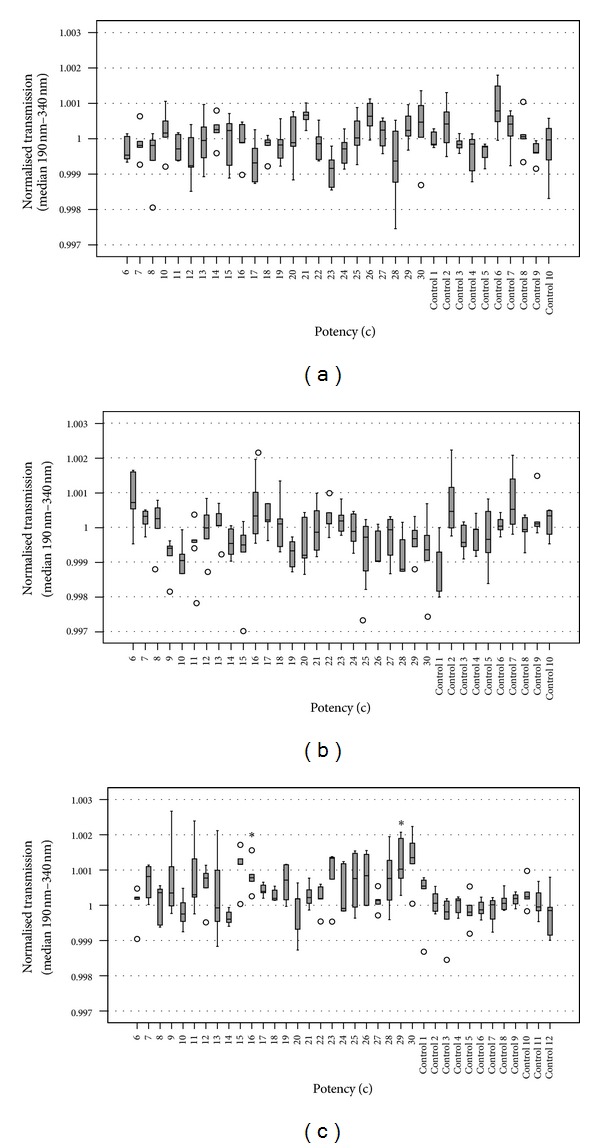
Boxplots showing light transmissions of potencies and controls (measurements 1 to 5) of CuSO_4_ (a), hypericum (b), S_8_ (c). Circles represent outliers (that lie more than one and a half box lengths above or below from the upper or lower quartile, resp.). Every potency level was compared to the combined controls using a Mann-Whitney-*U* test. Statistically significant results are marked by *. Due to multiple testing, *P* was corrected according to Bonferroni (*P ≤ 0.05/25 = 0.002).

Potency levels 6c to 30c of non-exposed hp were also compared among one another. Figures 2(a), 2(b), and 2(c) show wavelike patterns of higher and lower transmissions for the dilutions of all 3 preparation series. Kruskal-Wallis tests yielded mostly significant differences for the potency levels (190 nm–340 nm: *P*(CuSO_4_) = 0.032, *P*(hypericum) = 0.008,  *P*(S_8_) = 0.009; 220 nm–340 nm: *P*(CuSO_4_) = 0.051, *P*(hypericum) = 0.014, *P*(S_8_) = 0.012). Jonckheere's test showed a tendency towards ascending medians with ascending potency levels for CuSO_4_ (190 nm–340 nm: *P* = 0.080; 220 nm–340 nm: *P* = 0.072) and a tendency towards descending medians for hypericum (190 nm–340 nm: *P* = 0.057; 220 nm–340 nm: *P* = 0.065). A significant trend was revealed for S_8_ with higher transmission values for higher potency levels (190 nm–340 nm: *P* = 0.015, *z* = 2.425, *r* = 0.222; 220 nm–340 nm: *P* = 0.028, *z* = 2.196, *r* = 0.201).

When every single potency level was compared to the respective controls by Mann-Whitney-*U* test, only 2 potency levels of the S_8_ series (16c, 29c) showed a significant difference to the controls after Bonferroni correction for multiple testing (Figure 2(c)).

### 3.6. Comparison of Previous Works

**Figure 3:**
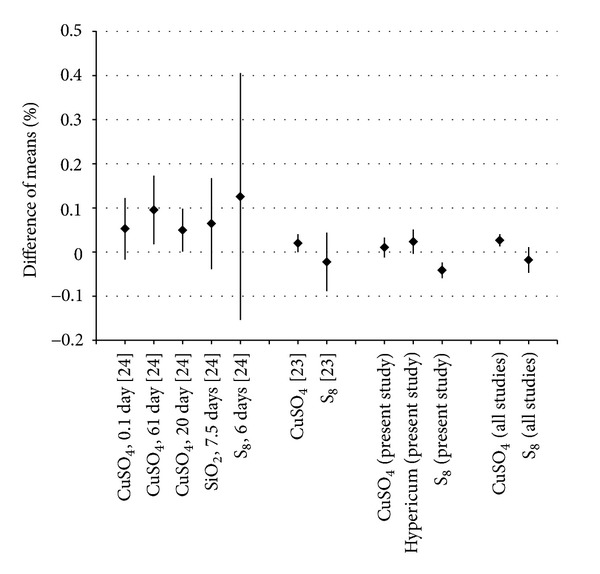
Differences of means (transmission of controls − transmission of hp) in % and 95% confidence intervals are shown. Previous and the present study are combined for CuSO_4_ and S_8_. The studies are weighed according to the number of measurement days, that is, 1 for [24], 6 for [23], and 5 for the present study.

Table 3 compares previous works of others and our group that investigated hp with UV, visible and/or near infrared light spectroscopy. In Figure 3, all results from our previous [23, 24] and present study are combined.

## 4. Discussion

### 4.1. Development of Light Spectroscopy Studies

The first studies that investigated hp with light spectroscopy compared whole spectra of hp and controls in mixtures of ethanol and water (Table 3). Zacharias [22] observed differences between hp prepared in pharmacies and under rigorous conditions of cleanness and concluded that changes in the spectra were caused by the introduction of contaminants during preparation. Rao et al. [19] found that the UV spectrum of succussed solvent (ethanol) differed from that of unsuccussed solvent. Korenbaum et al. [17] (comparing homeopathic nosodes and placebos) applied statistical tests in their comparisons and registered distinct wavelengths with significant differences between nosodes and placebos. Works from our group [23, 24] introduced series of hp, from 10c to 30c, and compared hp to succussed controls or different potencies of the same original substance. We used water as the solvent and no longer visually compared whole spectra, but applied statistical tests.

### 4.2. Reproducibility of Our Experiments

Difficulties in reproducing experimental results are sometimes used as arguments against specific actions of hp. In the present study, we investigated UV transmissions of hp for the third time. In the first study, hp of CuSO_4_ were found to have significantly lower UV transmissions than controls [24]. In the second study, slightly aged but not fresh hp of CuSO_4_ were found to have significantly lower UV transmissions than controls [23]. No differences were found between decimal serial dilutions of S_8_ and controls in either of these two studies.

For the present (third) study, modifications in the experimental setup and data analysis were made: the samples were not measured immediately after production (because in earlier studies, no significant differences could be observed at that time point), centesimal instead of decimal dilutions of S_8_ were prepared (to enable a comparison between different substances but of the same dilution category), and non-parametric statistical tests were used. Now significant differences between hp of S_8_ (but not of CuSO_4_) and controls were found. In both the second and third study, incubation to 37°C for 24 h led to differences between hp of CuSO_4_ and controls, and exposure of hp to 37°C or UV radiation led to reduced transmissions compared to non-exposed hp.

Hp of S_8_ had higher transmissions than controls, unlike hp of CuSO_4_ and hypericum. In the present study, we investigated centesimal potencies of S_8_ (S_8_c) in contrast to decimal potencies in earlier studies in order to be able to compare centesimal potencies of different starting substances. Therefore, it may well be to that we obtained different results because S_8_c may exhibit different features than S_8_x. In fact, in clinical use for some substances, such as sulfur and phosphor reciprocal effects depending on the potency level are known. If lower transmission was an indicator of a less structured state, higher transmission could be an indicator of a more structured state of the S_8_ hp. Additionally, in one of our previous studies [23], S_8_ also showed a different behaviour than CuSO_4_: when exposed to external factors, the variance of CuSO_4_ hp was increased, whereas the opposite was the case for S_8_.

Overall, hp and controls showed comparable differences in these three studies, indicating specific characteristics of hp. When these studies are combined, hp of CuSO_4_ have significantly lower transmissions than controls. Heat and ageing seem not only to change the physical properties of hp, but also their efficacy, as observed in a wheat germination model [37].

### 4.3. Possibility of Contaminations in hp

Earlier publications by other groups suggested contaminations to occur during the potentisation process [21, 22, 38]. In one of our previous studies, however, we showed that hp can be prepared with a minimum of inorganic contaminants, and differences in transmission of hp and controls are not due to contaminants [24]. According to the conclusions of a previous study [30], importance was attached in the experiments presented in this article to the cleaning of the bottles, the preparation of hp and controls (handling under a laminar flow, potentisation with water only, controls were succussed but not potentised) as well as the storage conditions (equal for hp and controls).

### 4.4. Models Assume Changes in Water Structure

So far, several models have been proposed to explain the different properties of hp and controls, including supramolecular states of dissolved gases and hydrogen-bonded supramolecular water structures [31] or dynamisation [30]. Most models assume the absence of traces of the starting material and focus on water structure, although it was reported that nanoparticles of metal starting materials may be found in high potencies [39]. Important questions remain how various starting materials can give rise to distinguishable physicochemical properties of the hp, for example the response to external physical factors that differed between CuSO_4_, hypericum and S_8_ in our experiments. It is a common criticism about homeopathic remedies, that if water had a memory of the original substances it came in contact with, it would be full of memories and would exert unpredictable effects [1, 4]. However, it has been shown that exposure of hp to external physical factors may reverse the properties of hp towards the properties of the solvents [31].

### 4.5. Limitations of This Work

Since the UV spectrophotometer was calibrated before each measurement series, there were small differences in the level of the absolute transmission values (in the order of <1%). These daily differences affected the controls and hp in the same way and are therefore not the reason for differences between controls and hp. As can be seen in Figure 2(a), not only the potency levels of CuSO_4_ showed variations in transmission, but also the controls deviated from each other. This may be the reason why the differences shown in previous studies between hp and controls of CuSO_4_ [23, 24] were not found in this study. Further differences were the person producing and measuring the hp and controls, as well as the location of the production and measurement. Exposures to external physical factors were done only once per factor (autoclave, EMF, incubation at 37°C, UV light) and per starting material (CuSO_4_, hypericum, S_8_) due to the limited total amount of our hp samples. In future studies repeating of exposure should be considered to obtain more indicative results. Additionally, it would be worthwhile investigating in future studies how repetitive exposure to physical factors would affect the results.

Trivial artefacts such as a cause for the differences between homeopathic preparations and controls can be ruled out due to the rigorous study design including randomisation and blinding of the samples.

## 5. Conclusions

This study confirmed and expanded some of our previous findings. By demonstrating differences in UV transmission between hp and controls, the study contributes to the understanding of physical properties of hp. It also shows that hp are not inert to for example heat and UV light and that their properties may change, which might be relevant for production, storage, and handling of hp.

## Figures and Tables

**Table 1:** Comparison^a^ between light transmissions of controls^b^ and potencies (6c–30c)^c^ of CuSO_4_, hypericum, and S_8_.

		CuSO_4_		Hypericum		S_8_	
		190 nm–340 nm	220 nm–340 nm	190 nm–340 nm	220 nm–340 nm	190 nm–340 nm	220 nm–340 nm
Measurement 1	Mean controls	0.999990	1.000002	0.999995	0.999999	0.999998	1.000001
	SD controls	0.000573	0.000539	0.000279	0.000258	0.000260	0.000246
	Mean hp	1.000037	1.000058	0.999826	0.999851	1.000093	1.000075
	SD hp	0.000637	0.000601	0.000349	0.000331	0.000279	0.000266
	*P*	0.910	0.955	0.160	0.171	0.299	0.363
	*r*	−0.019	−0.010	−0.238	−0.232	−0.171	−0.149

Measurement 2	Mean controls	0.999996	0.999998	1.000004	1.000001	1.000010	1.000006
	SD controls	0.000311	0.000311	0.000393	0.000371	0.000594	0.000578
	Mean hp	1.000062	1.000058	1.000005	1.000053	1.000648	1.000583
	SD hp	0.000500	0.000465	0.000460	0.000431	0.000676	0.000639
	*P*	0.791	0.806	0.596	0.488	**0.012**	**0.013**
	*r*	−0.045	−0.042	−0.090	−0.117	−0.411	−0.408

Measurement 3	Mean controls	0.999997	1.000007	1.000003	1.000020	1.000014	1.000010
	SD controls	0.000383	0.000338	0.001094	0.000984	0.000493	0.000478
	Mean hp	0.999810	0.999817	0.999663	0.999662	1.000684	1.000602
	SD hp	0.000492	0.000422	0.000955	0.000893	0.000610	0.000576
	*P*	0.449	0.241	0.454	0.476	**0.004**	**0.005**
	*r*	−0.130	−0.201	−0.127	−0.120	−0.475	−0.464

Measurement 4	Mean controls	0.999992	1.000000	0.999999	0.999997	0.999997	0.999991
	SD controls	0.001013	0.000905	0.000549	0.000484	0.000365	0.000366
	Mean hp	0.999624	0.999670	0.999660	0.999721	1.000506	1.000478
	SD hp	0.000896	0.000791	0.000780	0.000682	0.000844	0.000782
	*P*	0.450	0.364	0.154	0.177	0.071	0.068
	*r*	−0.130	−0.156	−0.241	−0.228	−0.297	−0.300

Measurement 5	Mean controls	1.000002	1.000009	1.000008	1.000008	0.999992	0.999994
	SD controls	0.000860	0.000792	0.001375	0.001247	0.000712	0.000659
	Mean hp	0.999926	0.999916	0.999673	0.999736	1.000170	1.000122
	SD hp	0.000800	0.000718	0.001312	0.001205	0.000793	0.000735
	*P*	0.985	0.821	0.701	0.688	0.973	0.864
	*r*	−0.003	−0.039	−0.065	−0.068	−0.006	−0.028

Autoclave	Mean controls	0.999994	0.999997	0.999993	0.999997	1.000002	1.000002
	SD controls	0.000629	0.000536	0.000557	0.000465	0.000519	0.000470
	Mean hp	0.999915	0.999943	0.999801	0.999802	1.000557	1.000497
	SD hp	0.000567	0.000517	0.000673	0.000587	0.000563	0.000520
	*P*	0.610	0.664	0.391	0.298	**0.011**	**0.014**
	*r*	−0.087	−0.075	−0.145	−0.176	−0.416	−0.403

EMF	Mean controls	1.000001	0.999998	1.000002	0.999996	1.000008	1.000002
	SD controls	0.000409	0.000376	0.000333	0.000302	0.000567	0.000527
	Mean hp	0.999765	0.999779	0.999845	0.999866	1.000661	1.000594
	SD hp	0.000475	0.000423	0.000588	0.000519	0.000715	0.000677
	*P*	0.198	0.219	0.913	0.942	**0.013**	**0.013**
	*r*	−0.221	−0.211	−0.019	−0.012	−0.408	−0.408

Incubation	Mean controls	0.999998	1.000003	1.000009	0.999997	0.999986	0.999997
	SD controls	0.000406	0.000371	0.000677	0.000632	0.001174	0.001122
	Mean hp	0.999629	0.999649	0.999999	1.000009	1.000649	1.000571
	SD hp	0.000431	0.000409	0.000383	0.000334	0.000623	0.000587
	*P*	**0.020**	**0.026**	0.289	0.298	**0.041**	0.115
	*r*	−0.399	−0.382	−0.179	−0.176	−0.336	−0.259

UV	Mean controls	1.000003	1.000005	1.000000	1.000000	1.000001	1.000009
	SD controls	0.000629	0.000612	0.000580	0.000569	0.000439	0.000413
	Mean hp	0.999610	0.999637	0.999558	0.999586	1.000676	1.000599
	SD hp	0.000342	0.000319	0.000536	0.000517	0.000545	0.000498
	*P*	0.212	0.281	**0.015**	**0.024**	**0.002**	**0.002**
	*r*	−0.214	−0.185	−0.411	−0.383	−0.520	−0.512

^a^by Mann-Whitney-*U* test, mean normalised transmission with standard deviation (SD) is shown, statistically significant results (*P* ≤ 0.05) are displayed in bold, *r* = effect size

^b^ *n* = 10 for CuSO_4_ and hypericum, *n* = 12 for S_8_

^c^ *n* = 24 for CuSO_4_ and *n* = 25 for hypericum and S_8_.

**Table 2:** Comparison^a^ between light transmissions of unexposed potencies (6c–30c)^b^ and potencies exposed to external physical factors^c^.

		CuSO_4_		Hypericum		S_8_	
		190 nm–340 nm	220 nm–340 nm	190 nm–340 nm	220 nm–340 nm	190 nm–340 nm	220 nm–340 nm
Unexposed	Mean	0.999936	0.999938	0.999834	0.999858	1.000666	1.000593
	SD	0.000507	0.000456	0.000761	0.000721	0.000637	0.000602

Autoclave	Mean	0.999915	0.999943	0.999801	0.999802	1.000557	1.000497
	SD	0.000567	0.000517	0.000673	0.000587	0.000563	0.000520
	*P*	1.000	0.738	0.857	0.669	0.451	0.590
	*r*	0.000	−0.039	−0.021	−0.049	−0.087	−0.062

EMF	Mean	0.999765	0.999779	0.999845	0.999866	1.000661	1.000594
	SD	0.000475	0.000423	0.000588	0.000519	0.000715	0.000677
	*P*	0.237	0.256	0.787	0.787	0.973	0.982
	*r*	−0.139	−0.134	−0.031	−0.031	−0.004	−0.003

Incubation	Mean	0.999629	0.999649	0.999999	1.000009	1.000649	1.000571
	SD	0.000431	0.000409	0.000383	0.000334	0.000623	0.000587
	*P*	**0.006**	**0.005**	0.536	0.629	0.902	0.857
	*r*	−0.322	−0.331	−0.071	−0.056	−0.014	−0.021

UV	Mean	0.999610	0.999637	0.999558	0.999586	1.000676	1.000599
	SD	0.000342	0.000319	0.000536	0.000517	0.000545	0.000498
	*P*	**0.001**	**0.001**	**0.029**	**0.033**	0.973	0.928
	*r*	−0.391	−0.386	−0.252	−0.247	−0.004	−0.010

^a^by Mann-Whitney-*U* test, mean normalised transmission with standard deviation (SD) is shown, statistically significant results (*P* ≤ 0.05) are displayed in bold, *r* = effect size.

^b^Measurements 2 and 3 were combined, since these two measurements were closest in time to the measurements of the exposed samples.

^c^ *n* = 48 for CuSO_4_ and *n* = 50 for hypericum and S_8_ (unexposed), *n* = 24 for CuSO_4_ and *n* = 25 for hypericum and S_8 _(exposed).

**Table 3:** Comparison of publications investigating homeopathic preparations with UV, visible and/or near infrared light spectroscopy.

Publication	Substances tested and controls	Methods	Findings
Ludwig, 1991 [18]	*Belladonna* (30x, 200x), 43% ethanol	Absorbance 190–220 nm comparison of spectra (no statistical analysis)	*Belladonna* 30x and 200x showed different UV spectra with a broader peak for 200x.

Zacharias, 1995 [21]	2 sets of samples of *Lycopodium clavatum* (6c, 12c, 100c), 40% water and ethanol mixture (unsuccussed, 3c, 6c)	Absorbance 220–800 nm (near zero beyond 400 nm) comparison of average spectra (5 spectra for each sample; no statistical analysis)	The spectra for each set of *Lycopodium* and succussed solvent were similar and differed from that of the inert solvent. The 2 sets of succussed *Lycopodium* samples showed significant differences. The possible introduction of contaminants during the dynamisation process was suggested.

Zacharias, 1995 [22]	3 sets of potentised hydroalcoholic solutions, 2 prepared in pharmacies (3c, 6c), one prepared under rigorous conditions of cleanness (3c, 6c, 9c, 12c)	Absorbance 220–800 nm (near zero beyond 400 nm) comparison of average spectra (5 spectra for each sample; no statistical analysis)	The dynamisation process caused changes in the UV absorption spectra of hydroalcoholic solutions prepared in homeopathic pharmacies, but not between unsuccussed and potentised solutions prepared under more rigorous conditions. It was concluded that the changes were caused by the introduction of contaminants during preparation of the samples.

Sukul et al., 2001 [20]	*Nux vomica* 30c (succussed and unsuccussed), 90% ethanol	Absorbance 190–500 nm comparison of spectra (no statistical analysis)	Unsuccussed *Nux vomica* 30 had its peak at 240 nm with an absorbance of 3.67, succussed Nux vomica 30 had one at 242 nm with an absorbance of 3.66. 90% ethanol had its peak at 206 nm with an absorbance of 2.23.

Korenbaum et al., 2006 [17]	7 homoeopathic nosodes (DNA-tox, bacteria, manus, fungus, toxic metal, virus, vanilmandelic acid) and a blank placebo were “imprinted” onto ampoules with saline.	Absorbance 600–800 nm centering of spectra, comparison of electronic-homeopathic copies (EHC) to every of the 3 placebo groups, registration of all wavelengths between 700–800 nm with significant differences, Mann-Whitney-*U* test	The spectra of each placebo group did not essentially differ from those of the other placebo groups. The spectrum of EHC manus differed significantly from all three placebo groups. The spectra of EHCs DNA-tox and toxic metal differed significantly from two placebo groups. The spectra of EHCs bacteria and vanilmandelic acid differed significantly from only one of the placebo groups. The spectra of EHCs fungus and virus did not differ from any of the placebo groups.

Rao et al., 2007 [19]	*Nux vomica, Natrum muriaticum* (6c, 12c, 30c in 95% ethanol), unsuccussed and succussed ethanol	Absorbance 200–500 nm comparison of spectra (no statistical analysis)	*Natrum muriaticum* and *Nux vomica* had different UV-spectra. The spectrum of unsuccussed ethanol was significantly different from that of succussed ethanol and the succussed homeopathic remedies, *Natrum muriaticum* and *Nux vomica*.

Wolf et al., 2011 [24]	SiO_2_ (10c–30c), S_8_ (11x–30x), CuSO_4_ (11c–30c), water succussed but not potentised	Transmission 190–290 nm, 215–290 nm mean transmission, correction for daily variations, *t*-test, ANOVA	UV transmission of CuSO_4_ hp (homeopathic preparations) was significantly lower than of controls. The transmission was also lower for both SiO_2_ and S_8_, but not significantly. The presence of contaminations was ruled out by inductively coupled plasma mass spectroscopy. An increase in the solvent's molecular dynamics for homeopathic preparations was suggested.

Marschollek et al., 2010 [23]	S_8_ (10x–30x), CuSO_4_ (6c–30c), water succussed but not potentised Samples were additionally exposed to UV light for 12 h, 37°C for 24 h or 90°C for 15 min.	Transmission 190–340 nm, 220–340 nm median transmission, correction for daily variations, *t*-test, Levene test	For CuSO_4_ (but not S_8_) lower UV transmission and higher variance was found for aged (26–110 days) hp compared to controls. Incubation of CuSO_4_ (but not S_8_) hp at 37°C resulted in significantly lower transmission compared to controls. For each type of exposure, transmission of CuSO_4_ hp was significantly reduced compared to unexposed hp. For S_8_, a significant reduction in transmission was observed after incubation at 37°C.

Klein et al., 2012 (present study)	S_8_ (6c–30c), CuSO_4_ (6c–30c), hypericum (6c–30c), water succussed but not potentised Samples were additionally exposed to UV light for 12 h, 37°C for 24 h, 90°C and pressure for 20 min or an electromagnetic field for 2 h.	Transmission 190–340 nm, 220–340 nm median transmission, correction for daily variations, Mann-Whitney-*U* test, Kruskal-Wallis test	Transmissions of controls and hp of S_8_ differed significantly on two of five measurement days and after exposure to physical factors. Transmissions of hp exposed to UV light and unexposed hp of CuSO_4_ and hypericum differed significantly. Potency levels 6c to 30c were also compared, and wavelike patterns of higher and lower transmissions were found with significant differences for potency levels of all three substances (as determined by Kruskal-Wallis test).
